# Evolution of quality characteristics and bacterial community in vacuum-packed soft-shell mud crab during slurry ice cooling and cold storage

**DOI:** 10.3389/fnut.2026.1762532

**Published:** 2026-02-13

**Authors:** Ye Sun, Shengming Han, Lei Liu, Changkao Mu, Chunlin Wang, Ce Shi, Yangfang Ye

**Affiliations:** 1 Key Laboratory of Aquacultural Biotechnology Ministry of Education, School of Marine Sciences, Ningbo University, Ningbo, China; 2 Ningbo Dasheng Biotechnology Co., Ltd., Ningbo, China

**Keywords:** bacterial community, cold storage, flavor, *K*-value, slurry ice, soft-shell crab

## Abstract

Slurry ice is known to extend the shelf life of refrigerated seafood, yet its specific impact on the quality and microbiota of soft-shell mud crabs during storage has not been well characterized. This study examined changes in the *K*-value, xanthine oxidase (XOD) activity, free amino acids, flavor nucleotides, and bacterial community succession in the muscle of vacuum-packed soft-shell crabs during slurry ice cooling and cold storage. The results showed that the crabs retained acceptable biochemical freshness (*K*-value < 40%) for up to 7 days. XOD activity increased over time. Gly, _L_-Arg, _L_-Ala, and _L_-Glu were identified as the predominant FAAs, and their marked reduction significantly influenced flavor development. Adenosine monophosphate was the most abundant flavor nucleotide, followed by inosine monophosphate; the decline in both compounds contributed to a reduction in the equivalent umami concentration. Furthermore, while the *α*-diversity of the muscle bacterial community remained stable, an increase was observed in four families—primarily Comamonadaceae and Shewanellaceae. Notably, the rise in amplicon sequence variant 936 (ASV936, assigned to *Shewanella*) by day 7 suggested the onset of spoilage. These findings demonstrate that slurry ice cooling combined with cold storage can effectively maintain the edible quality of soft-shell crabs for 7 days, offering a viable method for short-term preservation.

## 1 Introduction

The mud crab (*Scylla paramamosain*) is a highly valued marine crab species appreciated for delicious taste and rich nutrients such as protein, amino acids, and calcium (1). However, its hard shell poses significant inconveniences for consumers during consumption. Throughout the whole lifespan of crabs, more than a dozen times of molting are required for the growth, development, reproduction, and appendage regeneration. Crabs cast the old exoskeleton or shell including gill, foregut, outer membrane of hindgut, and residual gut content (2, 3). The crabs with incompletely hardened exoskeleton are called soft-shell crabs (4). Soft-shell crabs are entirely edible and contain more calcium and less fat than hard-shell crabs, rendering them one of the most commercially promising products in the mud crab industry (5–7). In general, the exoskeleton hardening of soft-shell crabs typically takes about 2 days. However, the period during which soft-shell crabs maintain their high commercial value is relatively short, averaging only 3 h (8, 9). In this circumstance, cold storage becomes a general method for the preservation of newly molted soft-shell crabs to maintain the softness of the shell. However, one thing we note that crabs are highly susceptible to spoilage due to enzymatic autolysis and microbial growth. Although low temperature can efficiently retard the spoilage process of mud crabs (10), the method to cool the crabs more rapidly needed to be further investigated and validated.

Slurry ice is a biphasic system containing small spherical ice particles and seawater, and has proven to be a useful technique in rapidly cooling aquatic products. Up to date, slurry ice has found widespread application in precooling and preserving fish (11–15), shrimp (16, 17) and shellfish (18). For example, the seawater slurry ice combined with electron beam irradiation is a better method to inhibit the activities of polyphenol oxidase, reduce the values of pH, total volatile base nitrogen, and total viable bacteria count, and maintain the good organoleptic quality of Pacific white shrimp (*Litopenaeus vannamei*) (19). Moreover, slurry ice using tea polyphenol-loaded chitosan/pectin nanoparticle as a nucleating agent also effectively extends the shelf life of large yellow croaker (*Pseudosciaena crocea*) based on the physicochemical indexes (20). As such, slurry ice seems to be effective to rapidly cool aquatic products; it is worthwhile to use it in the cold storage of soft-shell crabs.

In this work, we comprehensively analyzed the evolution of *K*-value, xanthine oxidase (XOD), free amino acids (FAAs), flavor nucleotides in the muscle of soft-shell mud crab during slurry ice cooling and cold storage. Meanwhile, we investigated the diversity and composition of muscle bacterial community using 16S rRNA gene amplicon sequencing. We aimed to reveal the following: (1) the evolution of quality characteristics in vacuum-packed soft-shell mud crab during slurry ice cooling and cold storage, (2) the succession of muscle bacterial community of vacuum-packed soft-shell mud crab, and (3) whether it is feasible for short-term preservation of vacuum-packed soft-shell mud crabs using slurry ice cooling and cold storage.

## 2 Materials and methods

### 2.1 Production of soft-shell crabs

A total of 60 double-shelled mud crabs with intact appendages (approximately 200 g each) were purchased from Aquatic Product Trading Market in Ningbo, Zhejiang, China. They were transported to the laboratory within 2 h using insulated foam boxes under dry conditions at an ambient temperature of approximately 25 °C. Each crab was housed individually in a plastic basket (30 cm × 19 cm × 15 cm) covered with a transparent acrylic sheet to prevent cannibalism between individuals and facilitate observation. Crabs were randomly allocated into six rearing tanks (1.5 m × 1.0 m × 0.6 m) with 300 L of natural seawater per each. The rearing seawater was constantly aerated and maintained at the following conditions: temperature 28–30 °C, pH 8.0–8.1, salinity 18–20‰, and dissolved oxygen 5–6 mg/L. A 30% daily water exchange was performed to maintain water quality. Crabs were not fed during the entire holding period to ensure clean water conditions for the subsequent soft-shell stage. Surveillance cameras were installed above the rearing tanks to timely obtain the newly molted crabs.

### 2.2 Determination of freezing point temperature

**Figure 1:**
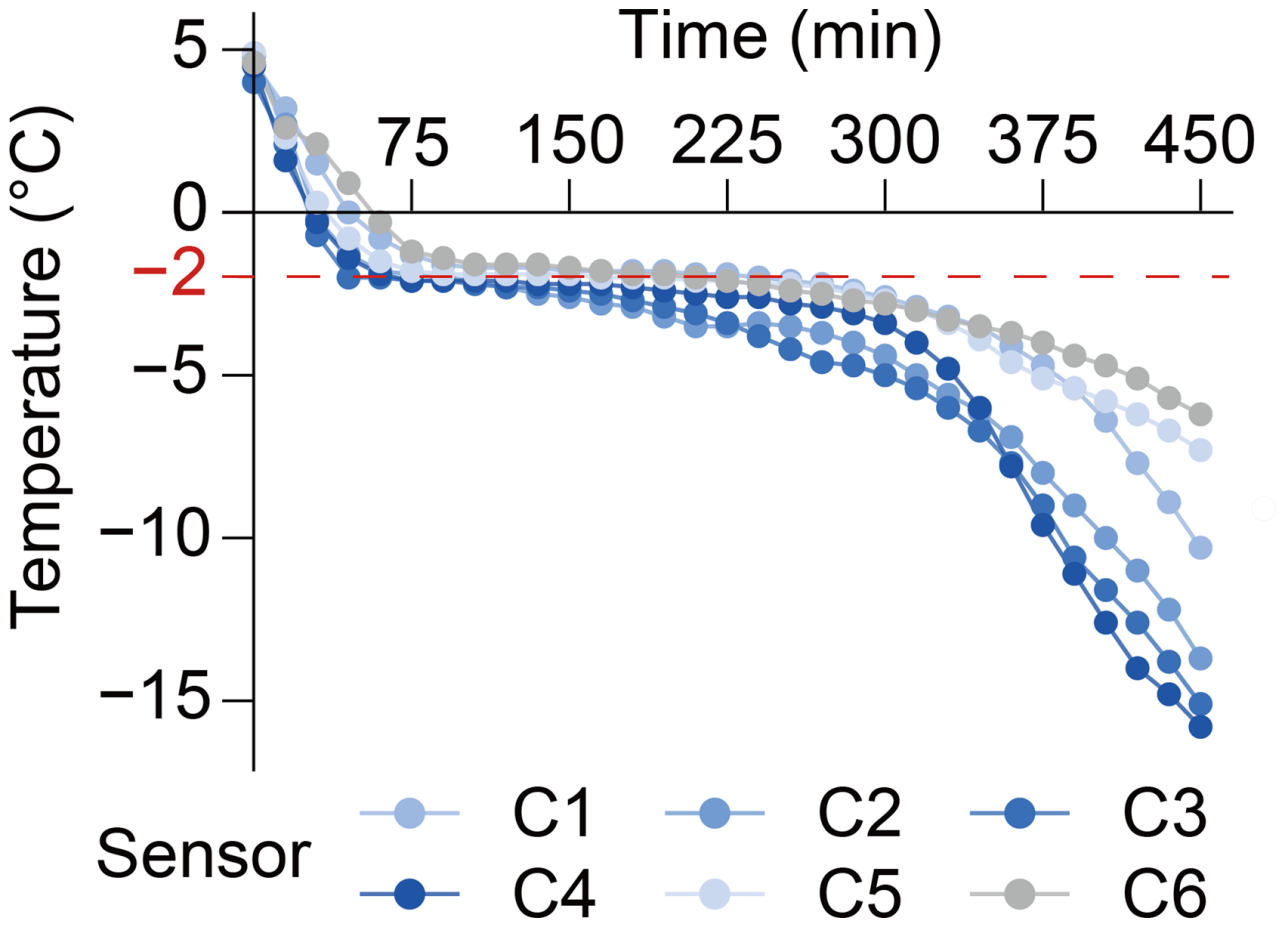
Freezing curves of soft-shell crabs. (C1–C6) Crab 1–crab 6.

Six soft-shell crabs were immobilized in an ice-water bath for 3 min. A probe from a mini temperature recorder (RC-4, Jiangsu Jingchuang Electric Co., Ltd.) was inserted into the cephalothorax of each crab, and the crabs were then placed in a −20 °C environment. Based on the freezing curves obtained from the six crabs (Figure 1), the freezing point temperature of the soft-shell crab was determined to be −1.5 °C.

### 2.3 Preparation of slurry ice

Natural seawater was cooled to its freezing point. Subsequently, titanium dioxide (TiO_2_) nanoparticles (anatase, 5 nm nominal particle size, Shanghai Aladdin Biochemical Technology Co., Ltd., China) were added as a nucleating agent at a concentration of 1% (w/w) of the total slurry ice mass. To achieve the final biphasic composition of 80% ice and 20% liquid seawater (by mass), the partially frozen seawater was vigorously agitated during the cooling process using a mechanical stirrer. The mixture was maintained in a low-temperature environment until a dense, flowable slurry with minimal free liquid was obtained. The temperature of the prepared slurry ice was continuously monitored and maintained at −1.5 ± 0.2 °C, verified using a calibrated temperature data logger (RC-4, Jingchuang Electric Co., Ltd., Jiangsu, China) with its probe immersed in the slurry center. This temperature aligns with the previously determined freezing point of the soft-shell crabs.

### 2.4 Slurry ice cooling and cold storage of soft-shell crabs and sample collection

**Figure 2:**
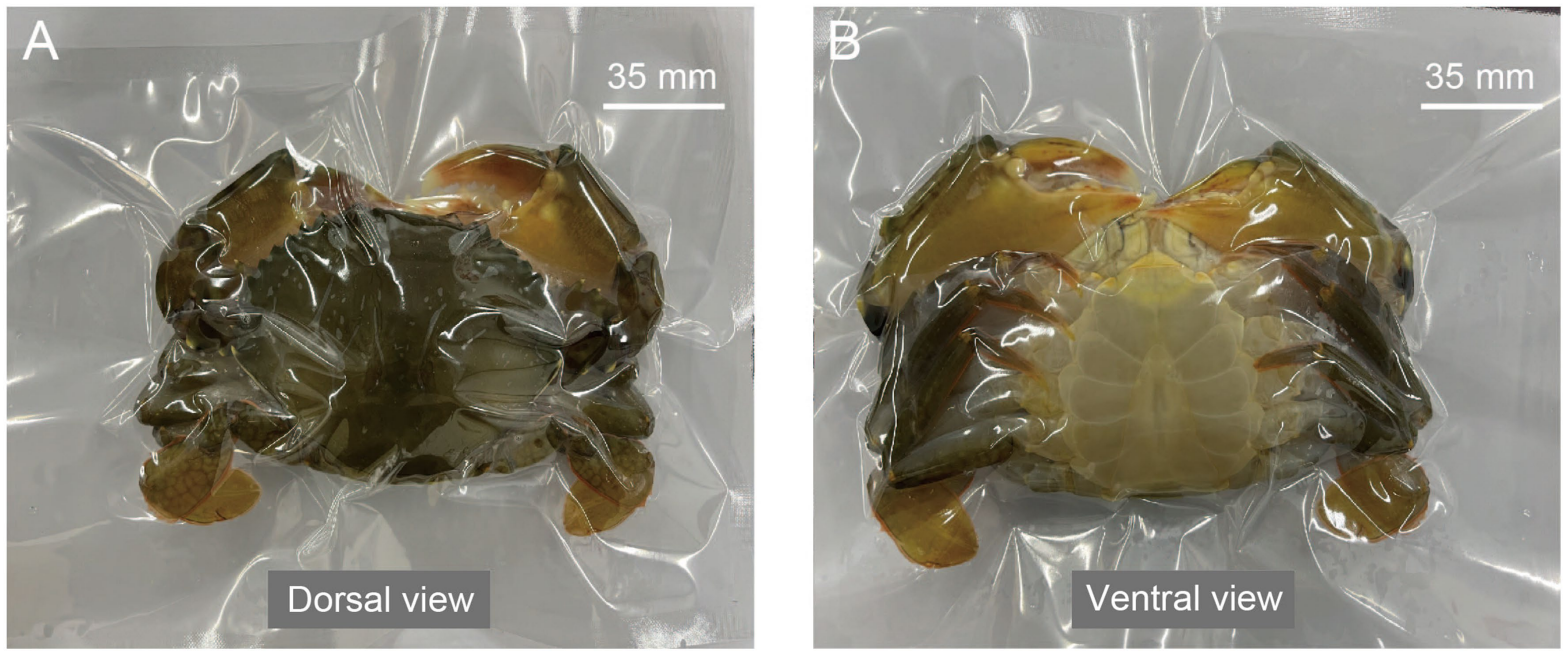
Vacuum-packed soft-shell crabs. **(A)** Dorsal view; **(B)** ventral view.

Thirty soft-shell crabs were selected and immediately anesthetized in an ice-water bath for 3 min. Six crabs were randomly designated as the control group (at day 0). The remaining 24 crabs were individually vacuum-packed using high-density polyethylene bags (Taizhou Hanyi Packaging Co., Ltd., Zhejiang, China) with a vacuum packaging machine (Model: HR-420, Xiamen Teliwei Equipment Co., Ltd., China). The machine settings were as follows: vacuum time was set to 27–30 s to achieve an internal vacuum of approximately 0.095 MPa, sealing time was 1.8–2.5 s, followed by a 4.0 s cooling cycle (Figure 2). HDPE was selected in this study to prevent direct contact between crabs and slurry ice owing to its effective barrier properties (21). Each vacuum-packed crab was then placed in a polypropylene (PP) plastic container (20 cm × 13 cm × 7 cm), completely embedded in slurry ice. A sufficient volume of slurry ice was used to ensure the crab was fully surrounded, with an approximate volume ratio of slurry ice to crab of 3:1. The containers were stored in a refrigerator at 4 °C. No renewal or supplementation of slurry ice was conducted during the 7-day storage period. All procedures and animal care were conducted in accordance with the Animal Research Institute Committee Guidelines for Ningbo University, China, and were approved by the Institutional Animal Care and Use Committee of Ningbo University. Crabs were sampled at specified time points (at days 1, 3, 5, and 7), with muscle tissues collected from six biological replicates (one crab per replicate) per time point. The muscle samples were specifically dissected from the thoracic appendage in the cephalothorax. Here, muscle tissue was selected for analysis because it is not only the primary edible part of crabs but also because its *K*-value serves as an optimal indicator of freshness in mud crabs (10). All samples were rapidly frozen in liquid nitrogen and stored at −80 °C until further analysis.

### 2.5 Enzyme assay

Muscle tissue samples (0.1 g) were homogenized in 0.9 mL of ice-cold 0.9% saline solution. The homogenate was then centrifuged at 3,000 rpm and 4 °C for 10 min. The resulting supernatant was collected for subsequent enzyme activity assays. The activity of xanthine oxidase (XOD) was determined using a commercial assay kit (Catalog No. A002-1-1 Nanjing Jiancheng Bioengineering Institute, China), according to the manufacturer’s instructions. Six biological replicates were performed.

### 2.6 Determination of *K*-value

*K*-value of crab muscle was measured using the paper electrophoresis method of a Freshness Checker (QS 3201, QS Solution, Japan) as described previously (22). Briefly, 1 g of muscle tissue was homogenized with 600 μL of extraction reagent A (provided with the kit). The homogenate was subsequently neutralized using extraction reagents B and C (also supplied by QS Solution). Thereafter, 3 mL of the resulting supernatant was subjected to paper electrophoresis, and the *K*-value was calculated accordingly.

It should be noted that the *K*-value, derived from this method, is a composite indicator based on the ratio of inosine monophosphate (IMP) and hypoxanthine to total ATP-related compounds. This study did not quantify the individual levels of adenosine triphosphate (ATP) and its direct precursors (e.g., ADP) throughout the degradation pathway.

### 2.7 Determination of amino acids

Amino acids were analyzed by ultra-high performance liquid chromatography–tandem mass spectrometry (UPLC-MS/MS) according to a method optimized for amino acid quantification (23) as detailed in a commercial analytical service report (Wuhan Metware Biotechnology Co., Ltd., China). In brief, approximately 10 mg of muscle tissue was weighed and mixed with 30 μL of amino acid internal standard solution (containing isotope-labeled analogs of all target amino acids). Then, 470 μL of ice-cold methanol–water (2:1, v/v) was added, and the mixture was extracted twice under vigorous vortex. The combined supernatant was derivatized with 5-aminoisoquinolyl-N-hydroxysuccinimidyl carbamate (5-AIQC) to tag the amino groups. After cooling to room temperature, 2 μL of formic acid was added. The solution was filtered through a 0.22 μm membrane and analyzed. Chromatographic separation was performed using an Agilent ZORBAX Eclipse Plus C18 column (2.1 × 100 mm, 1.8 μm; Agilent Technologies, Santa Clara, CA, United States) maintained at 35 °C. The mobile phase consisted of (A) water containing 0.004% (v/v) formic acid and 5 mM ammonium bicarbonate, and (B) methanol containing 0.16% (v/v) formic acid and 2 mM ammonium formate. A gradient elution was applied at a flow rate of 0.4 mL/min as follows: 5% B (0–2 min), increased to 20% B (2–5 min), to 35% B (5–6 min), held at 35% B (6–8 min), increased to 40% B (8–9.8 min), held at 40% B (9.8–10.4 min), increased to 95% B (10.4–11.1 min), and held at 95% B (11.1–14 min). Mass spectrometry detection was carried out on an Agilent 6,470 triple quadrupole mass spectrometer with an electrospray ionization (ESI) source in positive ion mode. The ion source parameters were set as: drying gas flow 10 L/min, drying gas temperature 315 °C, nebulizer pressure 50 psi, sheath gas temperature 350 °C, sheath gas flow 10 L/min, capillary voltage 4,000 V. Quantification was performed using MassHunter Workstation Software (v. B.08.00, Agilent Technologies, United States).

### 2.8 Determination of flavor nucleotides

Flavor nucleotides were determined by high-performance liquid chromatography (HPLC) following the method of Shang et al. (24) with slight modifications. Approximately 250 mg of muscle tissue was homogenized in 1.5 mL of 10% perchloric acid and sonicated for 10 min. The homogenate was centrifuged at 12,000 rpm and 4 °C for 10 min, and the supernatant was collected. The residue was re-extracted following the same procedure. The combined supernatants were diluted to 5 mL with a methanol–water solution containing 0.05 mol/L phosphoric acid and filtered through a 0.22 μm syringe filter. Nucleotide separation was carried out using an Alliance e2695 HPLC system (Waters, United States) equipped with an XBridge C_18_ column (4.6 mm × 250 mm, 5 μm). The column temperature was maintained at 40 °C, and the flow rate was 0.2 mL/min. Mobile phase A was 0.05 M potassium dihydrogen phosphate buffer (pH 6.5), and mobile phase B was methanol. An isocratic elution with 100% A was used for 15 min. Detection was performed at 254 nm using a UV detector.

### 2.9 Taste activity value (TAV) and equivalent umami concentration (EUC)

The TAV was calculated as the ratio of the concentration (C) of a flavor compound to its corresponding taste threshold (T), i.e., C/T. Compounds with a TAV ≥ 1 were considered to contribute significantly to flavor (25, 26). The taste threshold (T) values used were: 30 mg/100 mL for glutamic acid (Glu), 100 mg/100 mL for aspartic acid (Asp), 50 mg/100 mL for adenosine monophosphate (AMP), 25 mg/100 mL for IMP, and 12.5 mg/100 mL for guanosine monophosphate (GMP), as reported in the literature (27).

The EUC was expressed as grams of monosodium glutamate (MSG) per 100 grams of sample (g MSG/100 g). It was calculated according to the following formula:


$$\sum a_{i}b_{i}+1218(\sum a_{i}b_{i})(\sum a_{j}b_{j})$$

Here, *a*_i_ refers to the concentration of aspartic acid (Asp) or glutamic acid (Glu); *b*_i_ refers the relative umami coefficient for Asp (0.077) or Glu (1); *a*_j_ refers the concentration of AMP, IMP, or GMP; *b*_j_ refers the relative umami coefficient for AMP (0.18), IMP (1), or GMP (2.3); 1,218 refers a synergistic interaction constant.

### 2.10 DNA extraction, 16S rRNA gene amplification, and Illumina sequencing

Genomic DNA was extracted from 500 mg of crab muscle tissue using an E. Z. N. A.^®^ soil DNA kit (Omega Bio-Tek, United States). All muscle tissue samples were stored at −80 °C after collection, and DNA extraction was performed within 2 weeks of sampling. The concentration and purity of the obtained DNA were assessed using a NanoDrop ND-2000 spectrophotometer. The hypervariable V3–V4 region of the bacterial 16S rRNA gene was amplified with the primer pair 338F (5′-ACTCCTACGGGAGGCAGCAG-3′) and 806R (5′-GGACTACHVGGGTWTCTAAT-3′) (28), both modified with dual-index barcodes. PCR amplifications were performed in a total volume of 25 μL containing 12.5 μL of 2 × Premix Taq (TaKaRa, Dalian, China), 1 μL of each primer (10 μM), 2 μL of template DNA (approximately 20 ng), and 8.5 μL of sterile distilled water. The thermocycling program was as follows: initial denaturation at 95 °C for 3 min; followed by 30 cycles of denaturation at 95 °C for 30 s, annealing at 55 °C for 30 s, and extension at 72 °C for 45 s; with a final extension at 72 °C for 10 min. To minimize amplification bias, PCR was carried out in triplicate for each sample. The resulting amplicons were purified, assessed for fragment size, quantified, and pooled in equimolar ratios. Sequencing was performed on the Illumina Nextseq2000 platform (Illumina, United States) to generate paired-end reads. After demultiplexing, raw sequences were quality-filtered using fastp (0.19.6) (29) and merged with FLASH (v1.2.11) (30). The resultant high-quality sequences were de-noised using DADA2 (31) plugin in the Qiime2 (32) (v 2020.2) pipeline with recommended parameters, which obtains single nucleotide resolution based on error profiles within samples. DADA2 denoised sequences are usually called amplicon sequence variants (ASVs). To minimize the effects of sequencing depth, the number of sequences from each sample was rarefied to 26,734. Taxonomic assignment of ASVs was performed using the Naive bayes consensus taxonomy classifier implemented in Qiime2 and the SILVA 16S rRNA database (v138).

### 2.11 Statistical analysis

All statistical analyses were performed using R software (v4.3.1). For most datasets (including microbial community data, enzyme activities, and other unspecified metrics), group differences were assessed using the Kruskal–Wallis test with Benjamini–Hochberg (BH) *p*-value correction for multiple comparisons, implemented through the agricolae package (33). The FAA data were analyzed using one-way ANOVA with the tidyverse package (34), followed by Duncan’s multiple range test to identify significant differences between control (day 0 group) and groups at other time points. Differences in bacterial community composition were evaluated using three non-parametric multivariate methods (MRPP, ANOSIM, and Adonis) based on Bray–Curtis dissimilarity, as implemented in the vegan package (64). Cluster distributions were visualized using principal coordinate analysis (PCoA). Indicator species analysis at the ASV level was conducted with the labdsv package (35), with statistical significance defined as *p* < 0.05. All visualizations were generated with the ggplot2 package. Data are presented as mean ± standard deviation (*n* = 6), and differences were considered significant at *p* < 0.05.

## 3 Results

### 3.1 Changes in *K*-value and XOD activity

**Figure 3:**
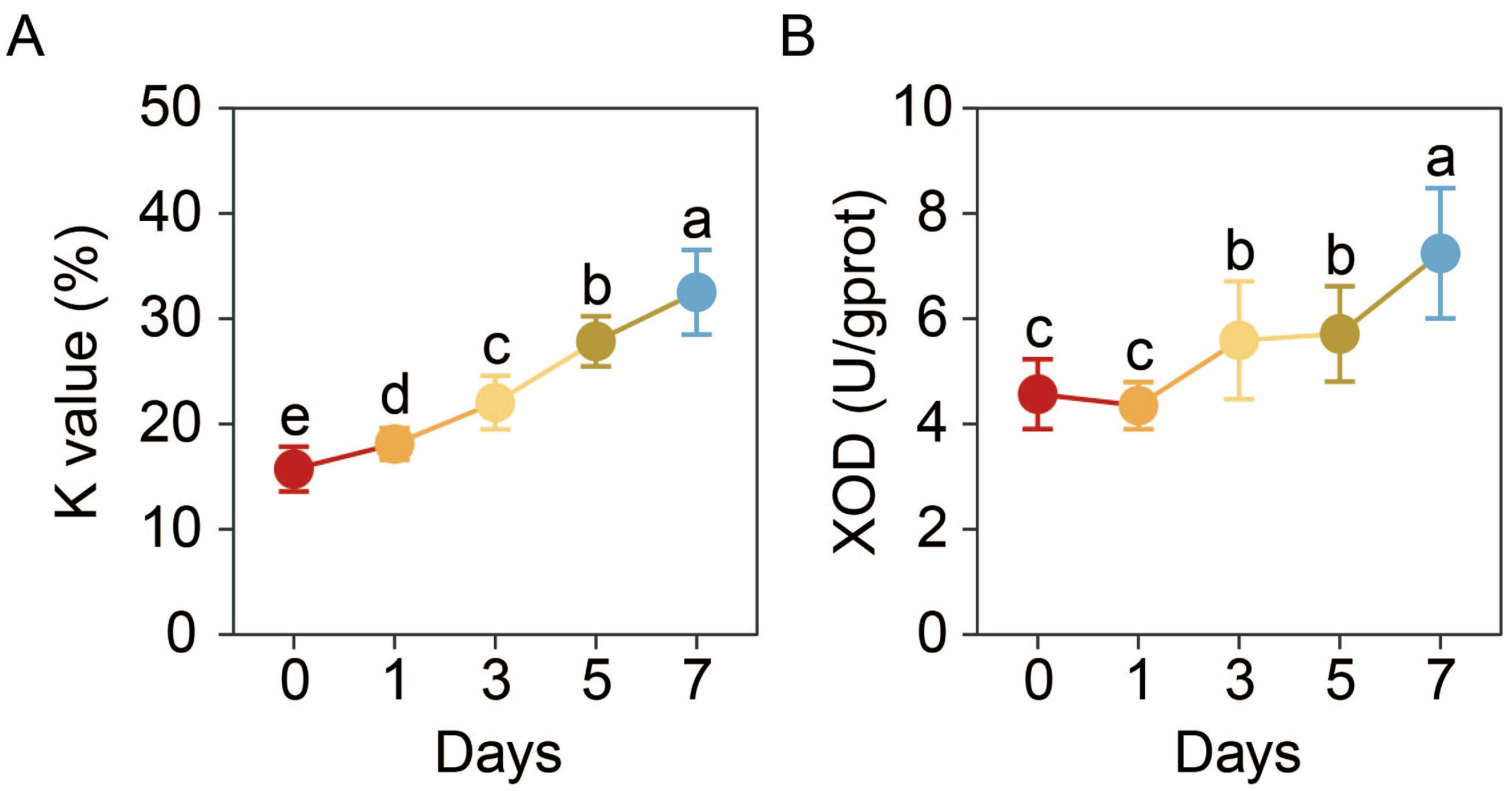
Changes in the *K*-value **(A)** and the activity of xanthine oxidase (XOD) **(B)** in the muscle of soft-shell crabs during cold storage. Different letters indicate a significant difference between groups (*p* < 0.05).

The muscle *K*-value was measured to evaluate changes in the freshness of refrigerated soft-shell crabs. The results indicated a continuous increase in the *K*-value throughout the cold storage period, rising from 15.72 ± 2.13% at day 0 to 32.52 ± 4.02% by day 7 (Figure 3A).

The muscle XOD activity of soft-shell crabs presented a significant increase at day 3 and another significant increase at day 7 (*p* < 0.05) (Figure 3B).

### 3.2 Changes in FAAs

A total of 20 amino acids were identified in the muscle of soft-shell crabs, including two umami amino acids (UAAs), five sweet amino acids (SAAs), 10 bitter amino acids (BAAs), and three tasteless amino acids (Table 1). Among the 17 FAAs, the concentrations of glycine (Gly) and arginine (_L_-Arg) exceeded 500 mg/100 g. Alanine (_L_-Ala) and glutamate (_L_-Glu) were present at levels of 283.96 ± 26.92 mg/100 g and 201.23 ± 78.48 mg/100 g, respectively. The concentrations of the remaining amino acids were predominantly below 25 mg/100 g, with the exception of proline (_L_-Pro, 53.44 ± 21.14 mg/100 g).

**Table 1:** Changes in the contents of free amino acids (FAAs) in the muscle of soft-shell crabs during cold storage.

FAA	Mean ± SD^a^ (mg/100 g)					Taste attribute	Taste threshold (mg/100 mL)	TAV				
	0 d	1 d	3 d	5 d	7 d			0 d	1 d	3 d	5 d	7 d
_L_-Glu	201.23 ± 78.48	136.84 ± 110.67	58.32 ± 33.63^*^	67.69 ± 31.82^*^	46.12 ± 6.93^*^	Umami	30	6.71	4.56	1.94	2.26	1.54
_L_-Asp	16.53 ± 11.60	13.74 ± 8.89	10.74 ± 8.09^*^	7.82 ± 1.29^*^	6.35 ± 1.05^*^	Umami	100	0.17	0.14	0.11	0.08	0.06
Gly	525.31 ± 75.90	311.89 ± 125.44	202.37 ± 22.92^*^	196.86 ± 36.71^*^	149.02 ± 34.54^*^	Sweet	130	4.04	2.40	1.56	1.51	1.15
_L_-Ala	283.96 ± 26.92	255.79 ± 253.54	130.89 ± 43.44^*^	173.72 ± 57.64^*^	133.95 ± 18.75^*^	Sweet	60	4.73	4.26	2.18	2.90	2.23
_L_-Pro	53.44 ± 21.14	58.40 ± 62.23	23.37 ± 10.09	34.32 ± 9.70	35.68 ± 11.69	Sweet	300	0.18	0.19	0.08	0.11	0.12
_L_-Thr	20.06 ± 4.41	21.40 ± 19.71	11.70 ± 5.49	13.81 ± 3.48	15.18 ± 2.71^*^	Sweet	260	0.08	0.08	0.05	0.05	0.06
_L_-Ser	8.85 ± 3.51	14.70 ± 16.08	13.93 ± 7.10	15.04 ± 7.56	14.37 ± 1.54^*^	Sweet	150	0.06	0.10	0.09	0.10	0.10
_L_-Arg	519.10 ± 155.86	329.35 ± 201.65	159.41 ± 20.23^*^	207.17 ± 80.93^*^	213.09 ± 90.69^*^	Bitter	50	10.38	6.59	3.19	4.14	4.26
_L_-Lys	24.78 ± 7.25	17.50 ± 18.36	17.15 ± 10.78	19.09 ± 4.59	21.42 ± 7.25	Bitter	50	0.50	0.35	0.34	0.38	0.43
_L_-His	23.30 ± 3.91	26.91 ± 30.95	10.59 ± 4.22^*^	13.59 ± 5.70^*^	15.06 ± 5.65	Bitter	20	1.17	1.35	0.53	0.68	0.75
_L_-Tyr	10.44 ± 3.85	19.88 ± 23.70	13.13 ± 6.56	11.94 ± 2.05	16.56 ± 3.94^*^	Bitter						
_L_-Leu	9.04 ± 2.19	11.35 ± 11.28	14.30 ± 8.25	12.89 ± 1.47^*^	18.72 ± 4.65^*^	Bitter	190	0.05	0.06	0.08	0.07	0.10
_L_-Phe	7.74 ± 3.10	10.95 ± 12.49	10.97 ± 6.95	8.97 ± 0.76	13.51 ± 3.80^*^	Bitter	90	0.09	0.12	0.12	0.10	0.15
_L_-Val	10.35 ± 4.23	14.16 ± 16.25	11.20 ± 6.89	10.25 ± 2.11	14.11 ± 3.74	Bitter	40	0.26	0.35	0.28	0.26	0.35
_L_-Met	11.39 ± 5.49	7.95 ± 7.95	7.89 ± 5.00	8.89 ± 2.89	9.12 ± 2.45	Bitter	30	0.38	0.27	0.26	0.30	0.30
_L_-Ile	4.60 ± 1.26	6.84 ± 7.60	8.31 ± 5.18	5.33 ± 0.66	7.98 ± 2.30^*^	Bitter	90	0.05	0.08	0.09	0.06	0.09
_D/L_-Trp	1.10 ± 0.80	1.01 ± 0.79	2.85 ± 1.75	2.42 ± 0.51	3.46 ± 1.33^*^	Bitter	200	0.01	0.01	0.01	0.01	0.02
_L_-Gln	232.00 ± 36.06	168.77 ± 95.34	77.83 ± 30.41^*^	144.29 ± 46.18^*^	125.29 ± 50.76^*^	Tasteless						
_L_-Asn	54.27 ± 9.58	50.39 ± 39.09	13.15 ± 6.18^*^	26.97 ± 7.65	18.40 ± 6.20^*^	Tasteless						
_L_-Cys	0.83 ± 0.21	1.77 ± 0.81	4.16 ± 1.62^*^	3.66 ± 0.82^*^	3.99 ± 0.51^*^	Tasteless						
UAAs	1027.02 ± 80.62	718.25 ± 478.53	402.31 ± 89.99^*^	446.09 ± 117.32^*^	335.43 ± 49.97^*^							
SAAs	891.61 ± 74.34	662.18 ± 470.30	382.25 ± 70.57^*^	433.73 ± 102.69^*^	348.19 ± 53.74^*^							
BAAs	621.82 ± 180.06	445.90 ± 325.16	255.79 ± 54.99^*^	300.55 ± 97.48^*^	333.03 ± 118.91^*^							
EAAs	112.35 ± 25.31	118.07 ± 125.02	94.95 ± 53.60	95.25 ± 18.11	118.56 ± 31.94							
TAAs	2018.29 ± 301.25	1479.59 ± 1039.66	802.24 ± 192.83^*^	984.72 ± 278.99^*^	881.36 ± 233.45^*^							

^a^Average concentration and standard deviation (mean ± SD, mg/100 g crab muscle) were obtained from six parallel samples. Significance when compared with 0 d: *p < 0.05. Glu, glutamate; Asp., aspartate; Gly, glycine; Ala, alanine; Pro, proline; Thr, Threonine; Ser, serine; Arg, arginine; Lys, lysine; His, histidine; Tyr, tyrosine; Leu, leucine; Phe, phenylalanine; Val, valine; Met, methionine; Ile, isoleucine; Trp, tryptophan; Gln, glutamine; Asn, asparagine; Cys, cysteine; UAAs, Umami amino acids; SAAs, sweet amino acids; BAAs, bitter amino acids; EAAs, essential amino acids; TAAs, total free amino acids.

The FAA composition profile was influenced by storage time, characterized by a notable decrease in the most abundant and representative FAAs including _L_-Glu, Gly, _L_-Ala, and _L_-Arg. By day 7, the content of _L_-Glu decreased significantly to 46.12 ± 6.93 mg/100 g (*p* < 0.05), accompanied by a decline in its TAV value from 6.71 to 1.54. Similarly, the contents of Gly and _L_-Ala decreased markedly, with the TAV values declining from 4.04 to 1.15 and from 4.73 to 2.23, respectively, over the storage period. Further, _L_-Arg also exhibited a substantial reduction in concentration, with its TAV decreasing from 10.38 to 4.26. Histamine (_L_-His) showed a decrease in TAV from 1.17 to 0.75. For all other amino acids, TAV values remained below 1 throughout the study. Correspondingly, the contents of UAA, SAA, and BAA decreased by 67.4, 61.0, and 46.4%, respectively. The total amino acid (TAA) content declined significantly from 2018.29 ± 301.25 mg/100 g to 881.36 ± 233.45 mg/100 g (*p* < 0.05). In contrast, the essential amino acid (EAA) content increased by 5.5% increase during the storage period.

### 3.3 Changes in flavor nucleotides

**Figure 4:**
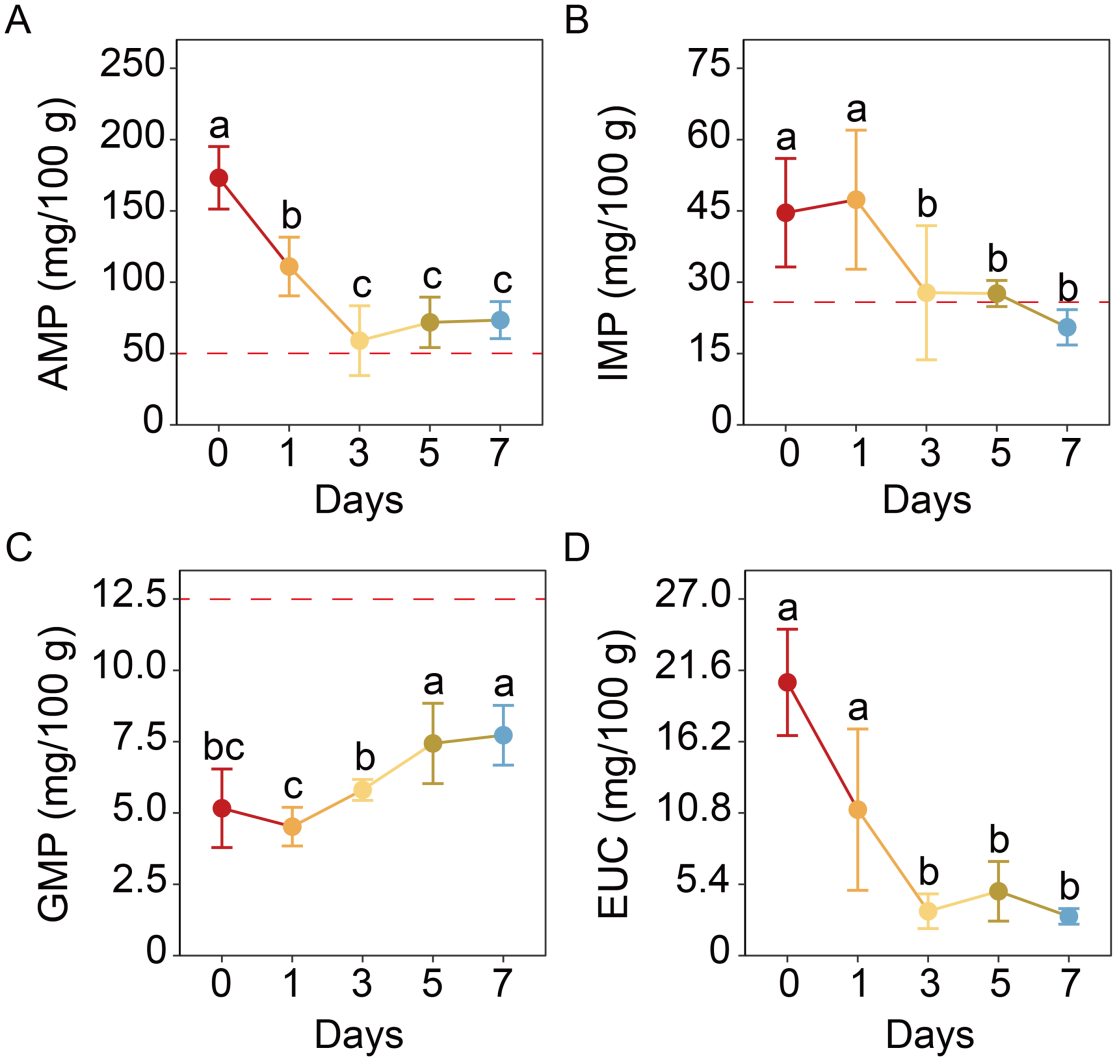
Changes in the flavor nucleotides including AMP **(A)**, IMP **(B)**, and GMP **(C)** as well as EUC **(D)** in the muscle of soft-shell crabs during cold storage. AMP, adenosine monophosphate; IMP, inosine monophosphate; GMP, guanosine monophosphate; EUC, equivalent umami concentration. The red dashed line represents taste threshold of flavor nucleotide. Different letters indicate a significant difference between groups (*p* < 0.05).

The AMP was identified as the most abundant nucleotide among AMP, IMP, and GMP in the muscle of soft-shell crabs (Figure 4A). Following refrigeration, its content exhibited an overall decline and became significantly lower than the initial level from day 3 onward (*p* < 0.05). IMP followed a trend similar to that of AMP, although no significant differences were detected during the entire storage period. In contrast, the content of GMP increased gradually and became significantly higher than the initial level starting from day 5 (*p* < 0.05) (Figures 4B,C). Meanwhile, the EUC of the muscle decreased by 85.65% over the 7-day storage period (Figure 4D).

### 3.4 Succession of bacterial community

**Figure 5:**
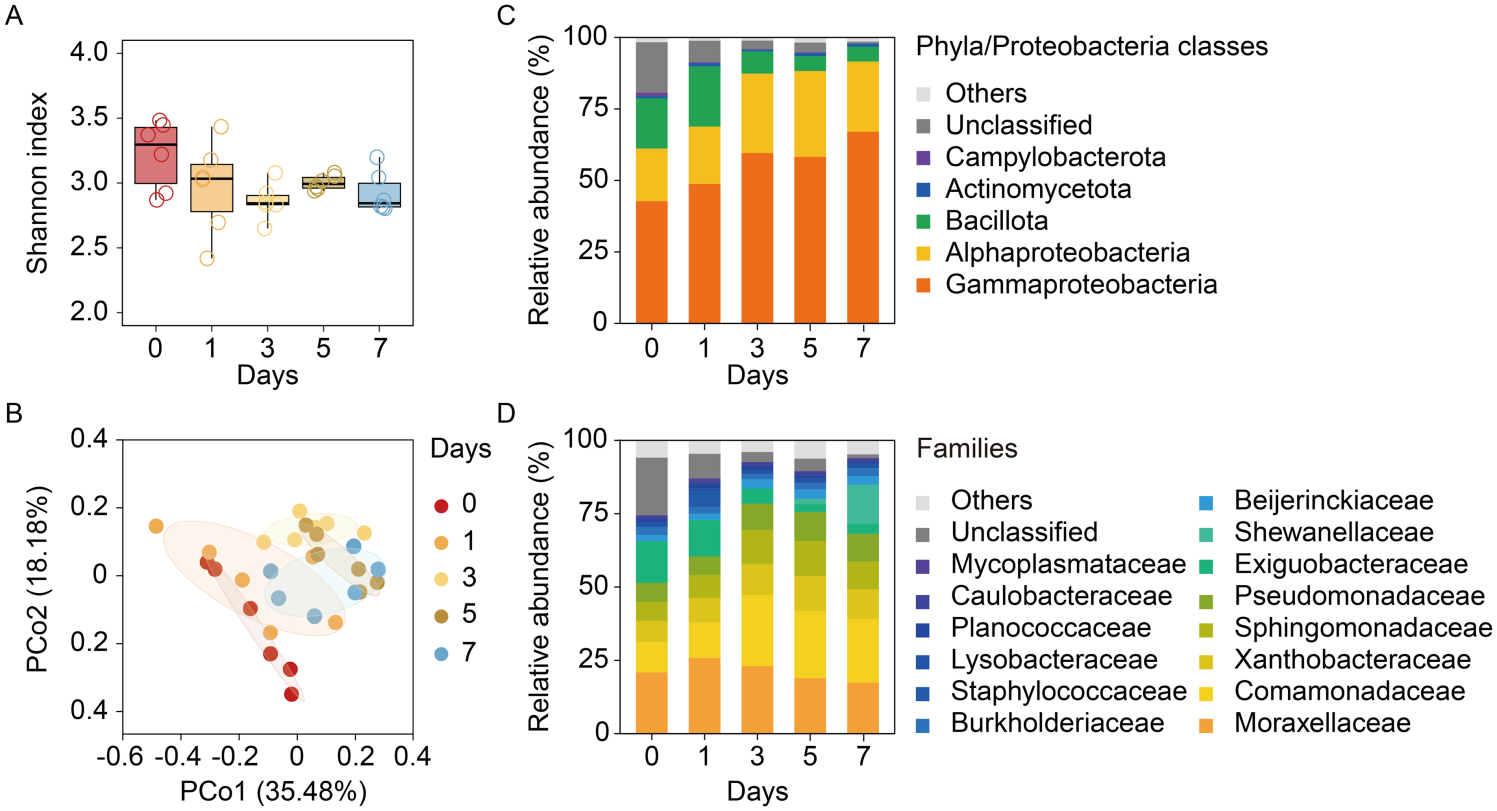
Changes in the bacterial community structure in the muscle of soft-shell crabs during cold storage. **(A)** Shannon index; **(B)** principal coordinate analysis (PCoA) plot based on Bray–Curtis dissimilarity visualizing compositional variations of bacterial communities with storage time; **(C)** dynamics of dominant phyla/proteobacterial classes with average relative abundance >1% at least in one group; **(D)** dynamics of dominant families with average relative abundance > 1% at least one group.

The Shannon index showed no significant differences across time points during storage (Figure 5A). PCoA analysis revealed a P-shaped trajectory in the succession of the bacterial community structure throughout the cold storage period (Figure 5B). Dissimilarity analyses indicated significant differences between crabs sampled at day 0 and those at days 3–7, as well as between crabs at day 1 and those at days 3–5 (*p* < 0.05, Table 2).

**Table 2:** Significance test of the differences in bacterial communities of soft-shell crabs between each refrigeration time point based on Bray–Curtis distance.

Group	MRPP		ANOSIM		ADONIS	
	δ	*p*	*r*	*p*	*F*	*p*
Day 0–Day 1	0.425	0.192	0.096	0.185	1.448	0.141
Day 0–Day 3	0.340	0.006	0.687	0.006	6.284	0.001
Day 0–Day 5	0.337	0.005	0.756	0.003	6.493	0.005
Day 0–Day 7	0.406	0.005	0.494	0.003	3.677	0.003
Day 1–Day 3	0.369	0.007	0.319	0.008	2.890	0.017
Day 1–Day 5	0.367	0.010	0.386	0.004	4.107	0.019
Day 1–Day 7	0.435	0.037	0.219	0.059	2.582	0.050
Day 3–Day 5	0.281	0.075	0.156	0.079	2.079	0.064
Day 3–Day 7	0.350	0.077	0.189	0.062	1.853	0.112
Day 5–Day 7	0.347	0.216	0.093	0.221	1.481	0.228

MRPP, multiple response permutation procedure; ANOSIM, analysis of similarity; Adonis, permutational multivariate analysis of variance.

During storage, the microbial composition in the muscle tissue shifted significantly. At the phyla/classes level, three dominant taxa changed over time: the relative abundances of Gammaproteobacteria and Alphaproteobacteria increased, whereas Bacillota decreased (Figure 5C). Changes were also evident at a finer taxonomic resolution, with more than 20 families (>1% in any group) showing altered relative abundances (Figure 5D); among these, Comamonadaceae and Shewanellaceae exhibited marked increases, with *Acinetobacter*, *Roseateles*, and *Shewanella*, among others, being the dominant genera in the resulting community (Supplementary Figure S1).

**Figure 6:**
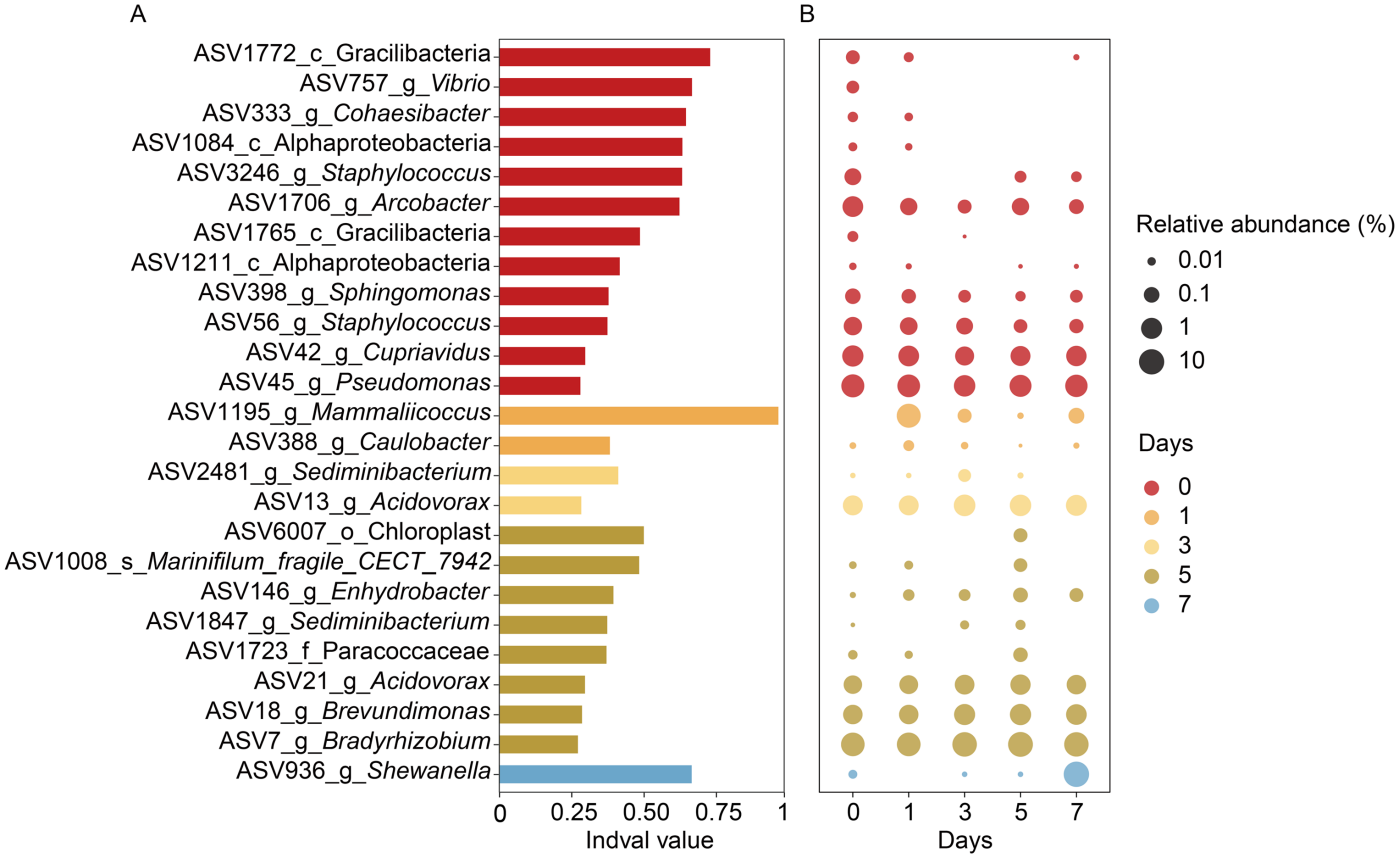
The values **(A)** and relative abundances **(B)** of ASV indicators in the muscle of refrigerated soft-shell crabs at day 0 (red), day 1 (orange), day 3 (yellow), day 5 (brown), and day 7 (blue). ASV, amplicon sequence variant; the lengths of the bars in plot A represent indicative values (Indval) of indicators. The diameters of the circles in plot (B) are proportional to the relative abundances of the ASVs, with the red, orange, yellow, brown, and blue circles indicating the peak relative abundances at five storage time points, respectively.

Given the distinct bacterial communities observed across different storage times, we further investigated key indicator ASVs associated with specific time points. A total of 25 indicator ASVs were identified (Figure 6). Nearly half of these indicators were most abundant in newly molted soft-shell crabs (day 0), such as two *Staphylococcus* ASVs (ASV56 and ASV 3246), one *Vibrio* ASV (ASV 757), and one *Arcobacter* ASV (ASV 1706) (Figure 6A). As storage proceeded, indicators ASV 1195 (assigned to *Mammaliicoccus*) and ASV 388 (*Caulobacter*) showed peak abundance at day 1 (Figure 6B). Two additional indicators ASV 2481 (*Sediminibacterium*) and ASV 13 (*Acidovorax*) exhibited the highest relative abundances at day 3. By day 5, eight ASVs dominated the muscle bacterial community, such as ASV 1008 (*Marinifilum fragile* CECT 7942) and ASV 1847 (*Sediminibacterium*). Notably, only one indicator ASV 936 (*Shewanella*) was prominent at day 7.

## 4 Discussion

### 4.1 Freshness reduction and textural deterioration during storage

The *K*-value is one of the most widely used biochemical indicators for assessing freshness of seafoods (36). In particular, muscle *K*-value serves as an optimal freshness marker in mud crabs, reflecting nucleotide degradation (10). In this study, the observed increase in *K*-value during storage aligns with typical post-mortem nucleotide breakdown. By day 3, the *K*-value reached 22.03 ± 2.55%. Although this value is comparable to values reported for Pacific white shrimp stored in seawater slurry ice (16), it did not meet the strictest freshness criteria for fish (*K* ≤ 20%). This elevated value may be attributed to the melting of TiO₂-based seawater slurry ice during storage at 4 °C, which could have compromised freezing stability. Despite that, the *K*-value remained below 40% through day 7, placing it within the “fresh level 1” range (20–40%) for fishery products and indicating acceptable edible quality (37). It should be noted that the *K*-value serves primarily as a biochemical reference for freshness evaluation. Practical assessment of edibility and safety must integrate microbial testing, sensory evaluation, and sanitary handling procedures. This supports the efficacy of slurry ice in extending the shelf life of seafood, as documented in numerous studies (11–15). Although the application of TiO₂ as a nucleating agent raises potential safety concerns regarding associated health risks (38), the detectable release of TiO₂ nanoparticles from food-contact materials into food matrices is typically minimal and considered negligible (39). Moreover, toxicological assessments conducted at doses far exceeding plausible exposure levels in our experimental setup have shown no significant health risks for food-grade TiO₂ (E171) (40). In addition, any potential indirect exposure pathway to crabs could be effectively mitigated by the HDPE barrier (21). Thus, the health risk posed by TiO₂ nanoparticles under our experimental conditions is very low and likely negligible. However, the polyethylene material may have slightly hindered heat transfer. Significant increases in XOD activity were observed at days 3 and 7 (*p* < 0.05; Figure 3B), marking critical phases for freshness loss. This aligns with the progression of ATP breakdown, in which XOD catalyzes the oxidation of hypoxanthine to xanthine and uric acid. The accumulation of xanthine is itself a well-established indicator of declining freshness (41–43). A limitation of this study is that the full ATP degradation pathway was not profiled. Future research employing techniques such as HPLC to systematically quantify ATP, ADP, AMP, IMP, and their degradation products would provide a more comprehensive understanding of the freshness evolution mechanisms in soft-shell crabs during chilled storage.

### 4.2 Flavor loss during storage

The freshness reduction of aquatic products is often accompanied by flavor loss (44). FAAs play a key role in flavor attributes such as umami, sweetness, and bitterness. A significant reduction in key FAAs—_L_-Glu, Gly, _L_-Ala, _L_-Arg—along with a decline in TAA content, indicates substantial flavor loss during storage. Notably, _L_-Arg, associated with bitterness, exhibited the most pronounced impact due to its high initial TAV and marked reduction. It is also noteworthy that SAAs such as Gly and _L_-Ala can contribute to umami flavor under high concentration conditions (45). To some extent, the amino acid profile observed in the muscle in this study differed from those reported in muscle or mixed muscle-hepatopancreas samples of soft-shell crabs (5, 6). These discrepancies may arise from variations in geographical origin (46), different developmental stage (47), and/or tissue specificity (25).

Nucleotides represent another major group of flavor-active compounds in mud crab muscle (25, 48). Given the high abundances of AMP and IMP in mud crabs (25, 46) and their synergistical interaction with amino acids to enhance umami perception (45), these two nucleotides along with GMP—another umami nucleotide (49) were selected to explore the changes in flavor nucleotides during cold storage. Our resulted showed that AMP was the most abundant among these three nucleotides in soft-shell crab the muscle, consistent with findings in hard-shell crabs (25, 46). IMP followed a trend similar to AMP, though no significant differences were detected throughout storage. A comparable decrease in AMP and IMP levels has been reported in refrigerated shrimp (16). Both AMP and IMP are known enhancers of the MSG-like taste, with taste thresholds of 50 mg/100 mL and 25 mg/100 mL, respectively (27). Consequently, both nucleotides contributed substantially to the overall flavor profile (TAV > 1). In contrast, GMP did not significantly influence flavor due to its content remaining below its taste threshold (12.5 mg/100 g).

It is important to note that umami nucleotides exhibit a synergistic effect in the enhanced umami flavor when combined with umami amino acids, quantifiable through the EUC. We observed an 85.65% decrease in the EUC of muscle over the 7-day storage period, a trend consistent with elevated EUC values reported in *P. trituberculatus* muscle under higher temperatures (50) and in edible tissues of *S. paramamosain* (48). Notably, the EUC values of soft-shell crab muscle were considerably higher than those of hard-shell crabs from seawater or saline-alkaline environments (25, 48, 51). Although the EUC decreased during storage, it remained higher than that of hard-shell crabs (25, 51), indicating that slurry ice cooling and cold storage helped preserve the umami flavor of soft-shell crabs for up to 7 days. However, it should be noted that while these chemical indices (EUC, TAV) are established predictors of sensory attributes, future studies should include direct human sensory evaluation to fully validate the taste quality and overall acceptability of the stored crabs.

### 4.3 Close relationships between bacterial community and quality

The proliferation of spoilage bacteria is a major driver of quality deterioration in seafood (52). Our microbial analysis provided a detailed examination of the bacterial community dynamics in soft-shell crabs over a 7-day storage period. While reduced bacterial *α*-diversity due to cold storage is commonly reported in products such as crab paste (53), monkfish (4), and large yellow croaker (54), the absence of a significant decrease in α-diversity in this study suggests that soft-shell crabs maintained a relatively diverse bacterial community throughout storage. This may be attributed to the use of slurry ice cooling. The P-shaped trajectory observed in the PCoA, along with significant dissimilarities at specific time points, reflects a dynamic ecological succession—though less pronounced than that reported in mud crab muscle or crab paste (10, 53).

Shifts in dominant bacterial phyla/classes and the increased abundance of specific families indicate microbial adaptation to storage conditions. Temporal changes in indicators further suggest a structured ecological succession within the muscle bacterial community. The reduced vacuum level and low storage temperature likely exerted selective pressure on bacterial survival, consistent with the principle of “survival of the fittest.” Notably, 12 indicator ASVs present in newly molted soft-shell crabs appeared poorly adapted to the vacuum and cold environment. Interestingly, an ASV identified as *Vibrio* (ASV757) was suppressed throughout storage. Although low temperature (4 °C) alone is generally insufficient to inhibit *Vibrio* growth (53), the vacuum packaging may have contributed to its suppression. This is consistent with previous findings in which vacuum-packaged oysters showed significantly lower levels of *V. vulnificus* compared to aerobically stored samples during frozen storage (55).

As storage progressed, no typical spoilage bacteria became dominant except for ASV936, assigned to the genus *Shewanella*, which increased markedly by day 7. *Shewanella* comprises approximately 70 Gram-negative species (56), among which *S. putrefaciens* is a known psychrotolerant spoilage organism in various seafoods, including fish (57) and shrimp (58). Low temperatures can promote biofilm formation in *Shewanella* species, enhancing their environmental adaptability and inhibiting competitors (59, 60). Spoilage associated with *Shewanella* has been documented in vacuum-packed seafood (61). Furthermore, *S. putrefaciens* has been shown to accelerate IMP degradation in vacuum-packed refrigerated large yellow croaker (*Larimichthys crocea*) fillets (62). Thus, an increase in *S. putrefaciens* abundance is a recognized indicator of declining seafood quality (63). The rise of ASV936 in this study suggests that spoilage had likely initiated in the soft-shell crabs by day 7.

## 5 Conclusion

The current study investigates the changes in quality characteristics and bacterial community composition in the muscle of vacuum-packed soft-shell crabs during storage with slurry ice cooling. The *K*-value remained below 40%, suggesting that the crabs retained acceptable freshness based on this biochemical index within the seven-day storage period. Concurrently, XOD activity increased over time. Gly, L-Arg, L-Ala, and L-Glu were identified as the most abundant and representative FAAs in the muscle. These four FAAs declined during storage and appear to have played a major role in shaping the flavor profile of the refrigerated soft-shell crabs. Among the flavor-related nucleotides, AMP was the most abundant. Both AMP and IMP showed an overall decrease in content throughout storage, yet contributed substantially to the overall flavor. Notably, the EUC also decreased with prolonged storage. In addition, the bacterial community in the muscle exhibited no significant shift in *α*-diversity, but an increase in the relative abundance of four families—primarily Comamonadaceae and Shewanellaceae. The rise in ASV936, classified as *Shewanella*, suggests that spoilage likely began by day 7. Together, these results indicate that slurry-ice cooling combined with cold storage provides a feasible and effective means of maintaining the edible quality of soft-shell crabs over 7 days of storage. Future work will include a direct comparison with conventional methods (e.g., flake ice) to better quantify the advantages of slurry-ice storage, while also focusing on identifying the key spoilage bacteria responsible for quality decline.

## Data Availability

The datasets presented in this study can be found in online repositories. The names of the repository/repositories and accession number(s) can be found: https://www.ncbi.nlm.nih.gov/, PRJNA1272269.

## Supplementary material

SUPPLEMENTARY FIGURE S1 Dynamics of dominant genes with average relative abundance > 1% at least one group.
