# Supplementary figures and images for: Evolution of quality characteristics and bacterial community in vacuum-packed soft-shell mud crab during slurry ice cooling and cold storage
Source: Front Nutr. 2026 Feb 13;13:1762532. doi: 10.3389/fnut.2026.1762532 (PMC12946150; doi:10.3389/fnut.2026.1762532)

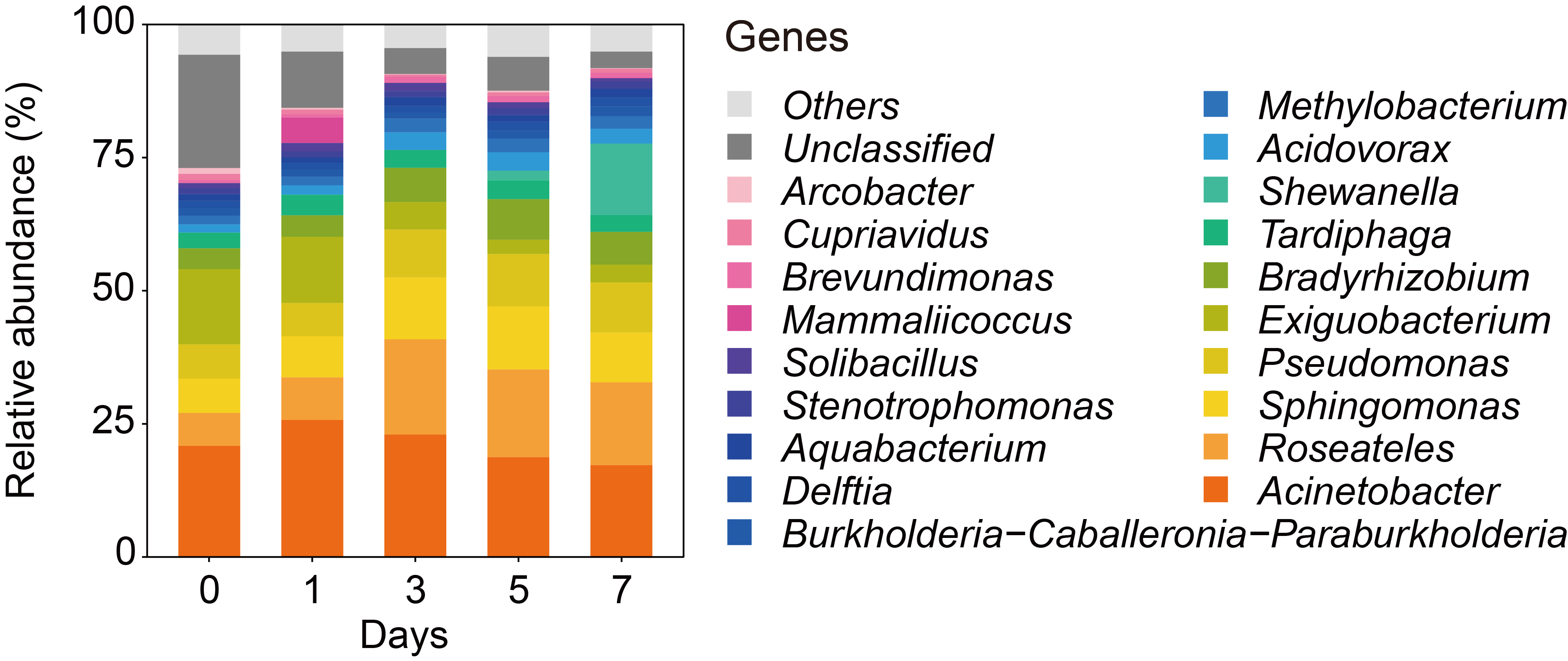

Supplement: SUPPLEMENTARY FIGURE S1 — Dynamics of dominant genes with average relative abundance > 1% at least one group. [file Image_1.png]
